# Effects of whole body vibration and backrest angle on perceived mental workload and performance

**DOI:** 10.17179/excli2020-2699

**Published:** 2021-02-18

**Authors:** Hamed Jalilian, Omid Gorjizadeh, Kamran Najafi, Mohsen Falahati

**Affiliations:** 1 Department of Occupational Health Engineering, Research Center for Environmental Pollutants, Faculty of Health, Qom University of Medical Sciences, Qom, Iran; 2 Student Research Committee, Shiraz University of Medical Sciences, Shiraz, Iran; 3 Student Research Committee, Hamedan University of Medical Sciences, Hamedan, Iran; 4 Department of Occupational Health Engineering, Faculty of Health, Saveh University of Medical Sciences, Saveh, Iran

**Keywords:** whole body vibration, backrest angle, mental workload, performance, mental task

## Abstract

Mental Workload (MWL) and human performance are widely contributing concepts in human factors. The objective of the current study is to investigate the perceived MWL and human performance during whole-body vibration (WBV) exposure while seated at different backrest angles. Nineteen healthy male participants completed both the NASA-TLX and rating scale mental effort (RSME) after performing two difficulty levels of computerized dual tasks. The participants' performance was measured in these conditions while seated with a backrest angle of 100 and 120 degrees and exposed to WBV (intensity: 0.5 m/s^2^; frequency 3-20 Hz) for 5 minutes. No significant effect on performance or perceived MWL (p<0.05) was found when changes were made to the backrest angles. Exposure to WBV under two backrest angles increased mental demand (p=0.04), effort (p=0.03) and frustration (p=0.03) and negatively affected human performance (p<0.05). The present study showed that exposure to WBV could be an important variable for designing work environments that require a high level of performance and mental demand while seated. However, the findings exhibited no association between inclining backrest angle and human performance or perceived MWL.

## Introduction

Mental workload (MWL) and human performance are widely contributing concepts in human factor and field research (Fallahi et al., 2016[12]; Faure et al., 2016[14]). Mental workload is defined as a theoretical construct representing the physiological and mental cost incurred by a human subject to achieve a specific level of performance (Hart and Staveland, 1988[20]). Human performance and MWL are mostly examined when ergonomists and system designers deal with the complexity of tasks, mental capacity, job demands, and in cases where an operator responds to unexpected events (Hancock, 1999[17]; Young et al., 2015[48]). 

The evidence indicates that drivers usually experience a high level of MWL when they perform routine driving tasks (Paxion et al., 2014[38]). Performance errors, more precisely MWL related problems, are responsible for the majority of road accidents (Davies and Underwood, 2007[6]; Dijksterhuis et al., 2011[8]; Hicks and Wierwille, 1979[21]). The literature suggests that the higher the level of MWL present, the higher the likelihood of actions resulting in risky behaviors will occur due to the lack of conscious awareness and a decrease in psycho-motor performance (Di Stasi et al., 2009[7]; Paxion et al., 2014[38]). However, there is still little evidence to describe the relationship between MWL levels and car accidents (Hoogendoorn, et al., 2010[22]; Wong and Huang, 2009[47]). Recent investigations have suggested a wide range of mental and physical stressors could elevate the MWL level among drivers and in turn, negatively affect human performance (Useche et al., 2018[46]). The evidence supporting the association between physical stressors as well as ergonomic risk factors and perceived MWL is not well documented (Newell and Mansfield, 2008[35]; Paddan et al., 2012[36]). Drivers are exposed to varying levels of whole-body vibration (WBV), noise, thermal conditions, and other ergonomic risk factors (Emkani et al., 2016[11]; Ismail et al., 2015[24]; Jamalizadeh et al., 2018[29]; Robb and Mansfield, 2007[40]). An experimental investigation reported that exposure to WBV could significantly reduce the performance of participants in a cognitive task and increase the perceived MWL (Khani Jazani et al., 2012[31]). Newell and Mansfield (2008[35]) found that exposure to WBV individually and in combination with other posture conditions (upright, twisted) could increase workload demand (Newell and Mansfield, 2008[35]). Using NASA task load index (NASA-TLX), Paddan et al. (2012[36]) reported a statistically significant increase in mental demands and resulted in poor performance among participants who completed a choice reaction time task under WBV exposure in different backrest angles. However, they did not find significant change while participants completed a tracking task under the same conditions (Paddan et al., 2012[36]).

Although many work environments demand mental load while sitting in exposure to the WBV, a few investigations have examined the overall effect on human cognitive and motor skills. These few findings indicated that performance in the motor-cognitive tasks might reduce and perceived MWL might increase when exposed to WBV. The current experimental study examines the human performance and perceived MWL during exposure to WBV at a frequency of 3-20 Hz and an intensity of 0.5 m/s^2^ in two common backrest angels of 100 and 120 degree. Therefore, the hypothesis of this study was that the exposure to the WBV and the reclined backrest angle when tested individually or in combination could change human performance and perceived MWL.

## Methods

### Participants

Nineteen healthy right-handed male university students (age: 24±4 years; BMI: 23±1 kg/m^2^) with a normal or corrected-to-normal vision volunteered to participate in this experimental study. The exclusion criteria included any history of diseases, alcohol and substance use, or abuse. Also, participants were asked to not drink coffee at least three hours prior to the experiment. Ethics Committee of Shiraz University of Medical Sciences approved the protocol of the study and each participant completed an informed written consent.

### Vibration simulator and monitoring

**Figure 1:**
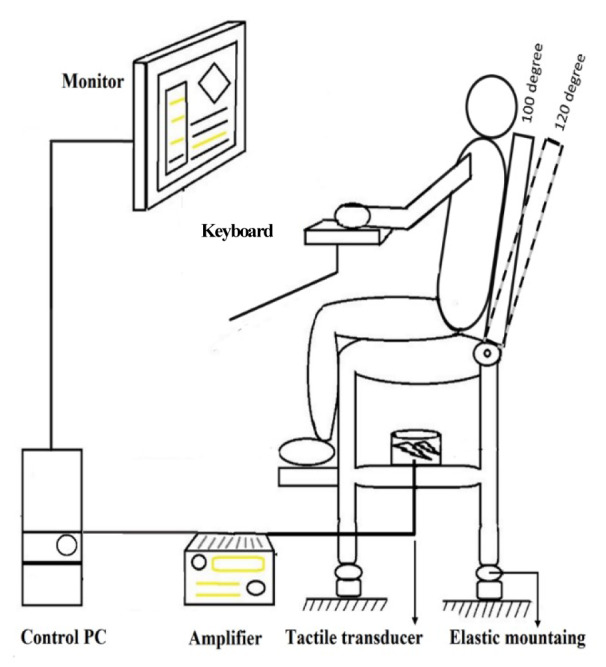
An illustration of vibration simulator when a participant using the experimental setup

A custom-made WBV simulator (Jalilian et al., 2019[28]) that included an adjustable car seat (Peugeot 405 driver seat, IK Co, Tehran, Iran) attached to a metal frame structure, a tactile transducer (ButtKicker LFE transducer, The Guitammer Co, Westerville, OH, USA) that was placed at the center of the metal structure, and an amplifier (ButtKicker BKA1000-N Power Amplifier, The Guitammer Co, Westerville, OH, USA) was utilized in this study (Figure 1(Fig. 1)). Vibration signals were generated by NI LabVIEW 2012 (National Instruments, Austin, TX, USA) and amplified to create three axial sine waves at different frequencies and intensities. 

Unweighted vibration was set to sine waves with a frequency of 3-20 Hz and an intensity of 0.5 m/s^2^ to simulate moderate vibration according to ISO 2631-1: 1997 (ISO, 1997[25]). The exposure of participants was monitored in real time using a SVAN 958 vibration analyzer with an SV 39A whole-body seat accelerometer (SVANTEK Sp. z oo, Warsaw, Poland), placed on the supporting seat surface (under ischial tuberosity).

A monitor and keyboard holder was set up with no connection to the WBV simulator and the hands of the participants had no contiguity with the simulator. Therefore, the monitor setup did not vibrate alongside with the metal frame and participants during the trials.

### Mental task and performance measure

A software was developed using C++ to create a dual task on the screen. The software offered two compensatory tracking tasks as well as a choice reaction time task to the participants, simultaneously. Various forms of test battery such as this have been used extensively in MWL studies (Paddan et al., 2012[36]; Ryu and Myung, 2005[41]). Participant's information such as age, education, marital status, gender, were entered before the test initiated. Participants were given maximum 1 minute to practice with the software and its functions. They had enough time to rest and to prepare for the study and then they could start the task by pressing a button once they were ready. 

**Figure 2:**
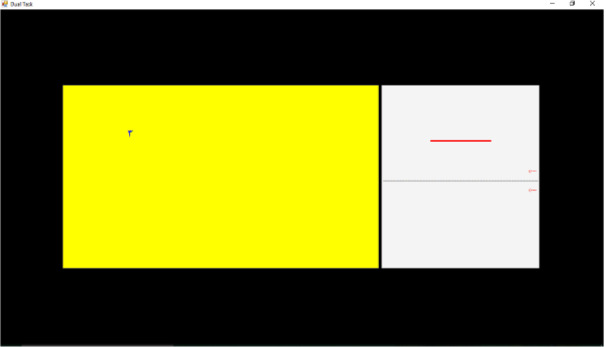
A snapshot of computerized dual task

Two separate tasks were created in the dual task page with dimensions of 1079*385 pixels. The tracking task on the right was 1/3^rd^ of the page (358*386 pixels) and the choice reaction time task on the left was 2/3^rd^ (716*386 pixels) of the screen to evaluate the participant's motor skills and mental workload (Figure 2(Fig. 2)). 

#### Tracking task

Tracking task was adapted from proposed dual task of International Organization for Standardization (ISO, 2012[26]). It included a dashed-black-horizontal line (center line) with the width of 1 pixel and length of 358 pixels that was positioned in the middle of tracking task, and a solid-black-horizontal line (target bar) with the width of 3 pixels and length of 137 pixels that was initially located over the center line before the participants start the software. Two red indicators were placed on the right of this screen, above and below the center line with the distance of 20 pixels. The target bar randomly left the center line in a positive or negative vertical direction, once the participants started. Participants were trained to control the target bar's movement by pressing the up and down keys on a keyboard in order to keep the target bar as close as possible to the center line (Jagacinski and Flach, 2003[27]; Paddan et al., 2012[36]). They knew that the target black bar would turn to red if it passed the arrow indicators on the right. 

The literature demonstrated that task demands (difficulty level of task) could change with reducing/increasing the speed of target bar, moved away from the center line in both direction (Paddan et al., 2012[36]; Ryu and Myung, 2005[41]). Therefore, a preliminary study was conducted to tune the difficulty of the task to the participants. Two task demands that were controlled by the speed of the target bar were selected. The movement speed of the target bar for the low speed and high speed were indicated as 80 and 160 pixels/second. Therefore, low task demand and high task demand were operationalized by presenting two speeds of 80 and 160 pixels/second, respectively. The results of the participants performance were extracted and are shown in Table 1(Tab. 1). 

#### Choice reaction time task

Choice reaction time task was adapted from proposed dual task of Paddan and colleagues (2012[36]) and Shieh and Lin (2000[44]), in which blue letters on a yellow background were shown to be the most preferable visual identification. Our design was customized so that a blue number (2, 3, 4, or 5) with the font of B Nazanin and size of 16 points was randomly shown to the participants while they had to respond to tracking task. They were instructed to press 'A' for numbers '2' or '3' and 'S' for '4' or '5' once they appeared on the screen. This generated four performance measures presented in Table 1(Tab. 1).

### Backrest angle

Two backrest positions were selected in two commonly recommended angles of 100 degrees and 120 degrees (Harrison et al., 2000[19]). A goniometer (Hi-Res 30, Sammons Preston Co, USA) was used to set the two backrest angles in all tests.

### Mental workload scale

Two subjective measures including NASA-TLX and rating scale mental effort (RSME) were used to assess perceived MWL in all tests. The NASA-TLX is a multi-dimensional scale based on six subscales including mental demands, physical demands, temporal demands, performance, effort, and frustration, which were scored from 0 to 100. The overall workload score is calculated based on the un-weighted (raw value) and weighted average of rating on the 6 subscales (Hart and Staveland, 1988[20]).

The RSME is a unidimensional scale consisting of a vertical line containing nine anchor points and running from 0 (“No effort at all”) to 150 (“Extremely effortful”). Using a mark on the line, the participant indicates the amount of mental effort taken to complete the task (Saris, 1988[42]).

### Procedure

All procedures were completed in the morning and in an environmentally controlled lab (noise level: 59±3 dB-A; temperature: 22±2 ºC; lighting level: 510±20 lux). Participants were instructed to complete the scales as well as the computerized dual task for 10 minutes following a 1-minute warm-up. Generally, 8 active trials were presented to each participant while seated (Table 2(Tab. 2)), in which they were asked to complete the mental tasks form at the end of each for 5 minutes (Figure 2(Fig. 2)) test. To control the order and sequence effects, all active tests randomly were presented to the participants. The scales were applied and completed immediately after each of the active tests and all performance measures were saved automatically. Participants were given 5 minutes to rest with open eyes, while they continued to sit on the simulator while it was off. Each participant generally was engaged in this experiment for 90 minutes.

### Data analysis

This exploratory study conducted for a problem that has not been studied more clearly or a few reports are available on the problem. Since it would be better to use a more liberal posthoc test (Armstrong and Hilton, 2010[1]) to do not miss a possible effect, i.e., to avoid a type II error (Armstrong, 2014[2]). Therefore, the researcher did not correct p-value for multiple testing. So that, the within-subjects and interaction effects of the independent variables (WBV, backrest angle and task demand) on dependent variables (performance measures and perceived MWL parameters) were assessed using repeated measure ANOVA followed by the least significant difference (LSD), a post hoc test to assess exactly which pairs differ significantly. Anyway, the mean effect of task demand variable on perceived MWL and performance were not reported here because enough evidence has been reported on these relationships. SPSS version 20 (SPSS Inc., Chicago, IL, USA) and GraphPad Prism version 7 (GraphPad Software, San Diego, CA, USA) were used for analyzing the data and graphical illustrations, respectively.

## Results

### Descriptive observation

**Figure 3:**
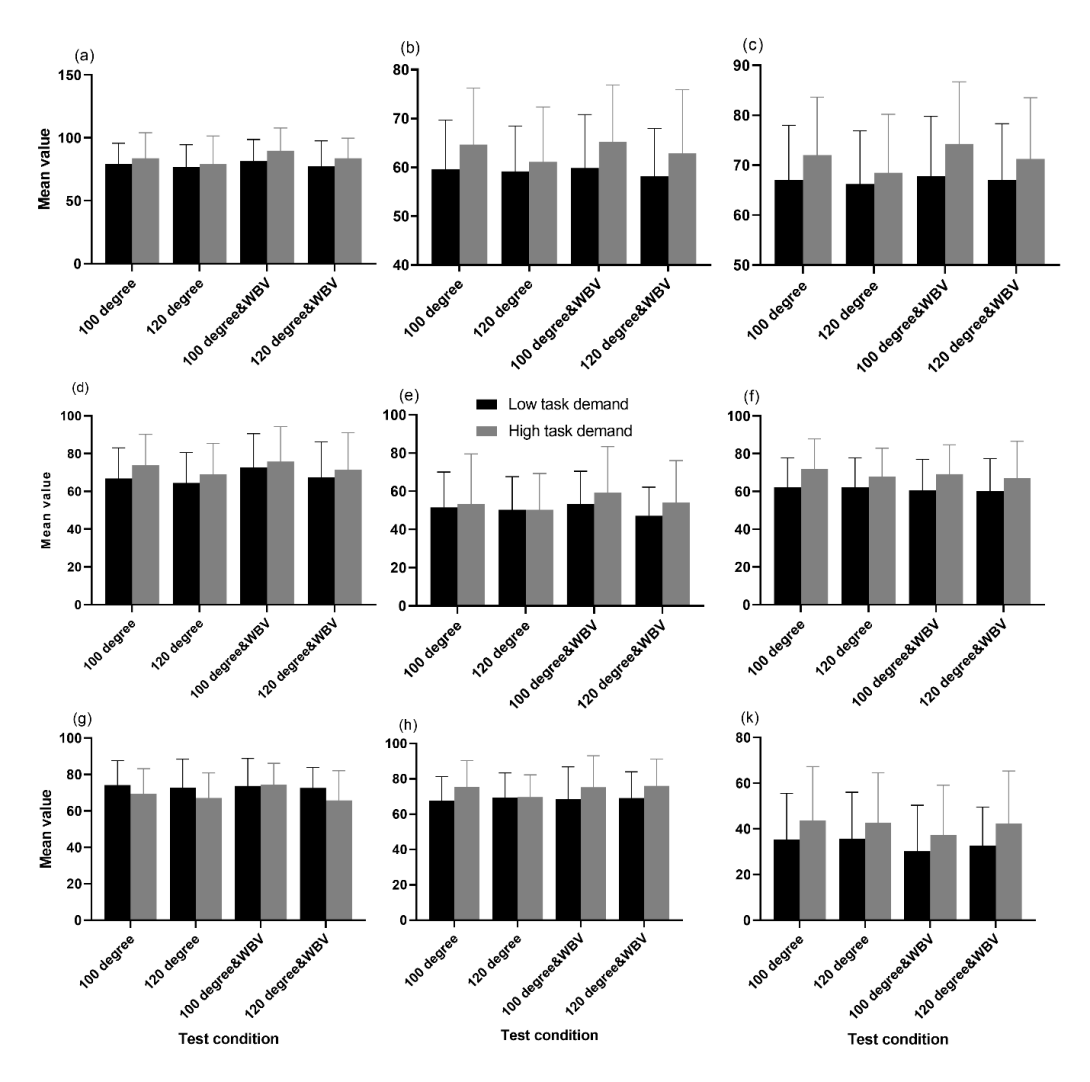
The mean rated score of (a) RSME, (b) raw NASA-TLX, (c) weighted NASA-TLX, (d) mental demand, (e) physical demand, (f) temporal demand, (g) performance level, (h) effort and (k) frustration among participants during different test conditions Note: The bar indicates standard deviation

Figure 3(Fig. 3) demonstrates the mean value of perceived MWL among 19 participants. Descriptive statistics demonstrated that in all test conditions, the RSME (Figure 3a(Fig. 3)) and NASA-TLX subscales (Figure 3d to 3k(Fig. 3)), scores were rated higher when subjects performed high task demand, except performance. Identical findings were observed when the raw and weighted NASA-TLX scores (Figure 3b and 3c(Fig. 3)) were calculated.

**Figure 4:**
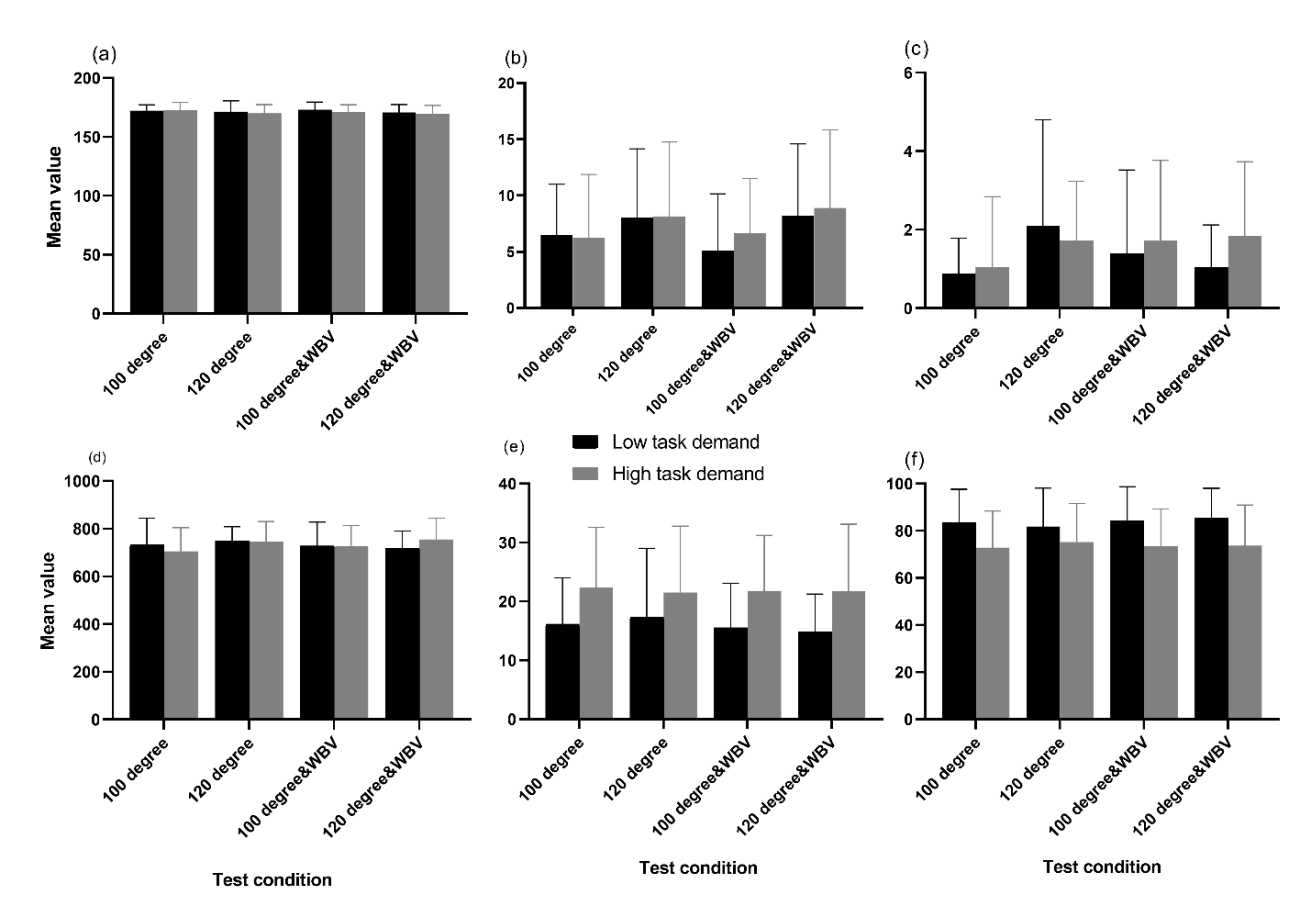
The mean scores obtained from tracing task measures including number of (a) correct, (b) incorrect, (c) missed answers, (d) reaction time (ms), chosen reaction time task measures including (e), mean distance of target bar from the center line (pixel), and (f) the time of being the target bar in the bounds of the center line during different test conditions (%). Note: The bar indicates standard deviation

Figure 4(Fig. 4) shows the mean value of participants' performance. The findings (Figure 4a and 4b(Fig. 4)) showed that the correct answers were higher at 100 degrees compared to 120 degrees and the incorrect answers were higher at 120 degrees compared to 100 degrees in both workloads. The results of the chosen reaction time task (Figure 4e and 4f(Fig. 4)) revealed that participants obtained higher performance while completing the low task demand compared to the high task demand.

### Effect of backrest angle

While participants completed the two levels of task demand without/under vibration exposure, changing the backrest angle from 100 degrees to 120 degrees had no statistically significant effect on the performance and the rated NASA-TLX subscales, raw and weighted scores, as well as the RSME score (p<0.05).

### Effect of whole-body vibration

The results of ANOVA test indicated a statistically significant difference between the within -subjects effects of WBV on mental demand (F (3,54) = 2.95; p= 0.04), effort (F (3,54) = 2.91; p= 0.04) and frustration (F (3,54) = 4.66; p= 0.006). The comparison of perceived MWL of the participants with and without vibration exposure under two backrest angles revealed a higher mental demand (p=0.04), effort (p=0.03) and frustration (p=0.03) while they were exposed to vibration (Table 3(Tab. 3)). In all conditions, the raw and weighted score of NASA-TLX as well as RSME score were rated higher under vibrational condition, but these relationships were not statistically significant. 

The findings of ANOVA test showed a statistically significant difference between the within -subjects effects of WBV on missed responses (F (3,51) = 2.97; p= 0.04), reaction time (F (3,54) = 2.93; p= 0.04), PT (F (3,54) = 16.41; p< 0.001) and MT (F (3,54) = 12.58; p< 0.001). Table 4(Tab. 4) indicated that the WBV exposure significantly increased the missed responses (p=0.04), reaction time (p=0.04), PT (p=0.03) and MT (p=0.04) compared to the unexposed condition.

### Interaction effects

The ANOVA indicated no significant interaction effects between the WBV, backrest angle and task demand regarding the dependent variables (all F(3,54) < 2; all p > 0.05).

## Discussion

The present study aimed to investigate whether the seat backrest angle and WBV could influence the perceived mental effort and human performance while participants were completing a dual task. This research showed that, in general, the level of performance was higher, but non-statistically significant, at the 100 degree angle compared to the 120 degree angle. Additionally, the findings revealed a significant effect of WBV on the perceived MWL and performance.

A few studies raised concerns regarding human performance and perceived MWL under different backrest angles, and have yielded inconsistent findings. Thody et al. (1993[45]) reported that the human performance measures including the reaction time and incorrect responses showed no statistically significant changes under three different backrest angles (7- 30- and 60-degree) (Thody et al., 1993[45]). The study by Edwards et al. revealed that detecting the target rate during 80 minutes increased by raising the backrest angle, but the detecting time was not affected significantly (Edwards et al., 1994[10]). Another study observed how the backrest angle significantly affected certain performance measures, but non-significant findings of perceived MWL, and the post hoc test demonstrated that 122 degrees backrest angle produced lower performance than 90 degrees. However, the performance measure calculating choice reaction time during the tasks was not affected by changing the backrest angle (Paddan et al., 2012[36]).

Generally, non-statistically significant findings in the current study as well as the inconsistent results of other papers regarding the human performance showed that inclining the backrest angle most likely has no effect on this variable, at least up to 120 degrees. These findings demonstrate that resting the back against an inclined back support transfers a considerable portion of the upper body weight to the backrest and decreases strain on the discs and muscles (Grandjean, 1986[16]). Therefore, chair designers have recommended an inclination of 110 degrees to 120 degrees for the backrest angle in order to create an optimum condition to mitigate risks concerning disc pressure and muscular activity (Grandjean, 1986[16]; Harrison et al., 2000[19]; Schmidt et al., 2014[43]). In addition to the physiological variables, perceptions of (dis)comfort for a seated subject could significantly affect human performance as well as perceived mental loads (Fan et al., 2020[13]). However, some evidence indicated that the comfort might be rated as highest in conditions that would not necessarily be considered biomechanically ideal (Carcone and Keir, 2007[3]). Overall, we observed a paucity of research on this topic and more investigations may be required to meet a conclusion. Therefore, it is felt that there is an urgent need for studies into the assessment of human performance and perceived MWL under different backrest angles and other ergonomic factors (e.g. arm rest angle).

In this study participants perceived a higher statistically significant level of mental demand, effort, and frustration while they completed the tasks under WBV exposure. Additionally, the main NASA-TLX and RSME scores were rated higher, but non-significant, under vibrational condition. Studying the influence of postures and multi-axis vibration on the perceived MWL revealed that all NASA-TLX dimensions were rated at a significantly higher amount by participants under vibrational conditions compared to the control condition (Newell and Mansfield, 2008[35]). Khani Jazani et al. (2012[31]) found that different intensities of vertical WBV could induce different levels of substantial mental demand (Khani Jazani et al., 2012[31]). Another study observed a general trend for an increase in the subjective workload as the vibration magnitude increased. Hancock et al. (2008[18]) demonstrated that vibration makes it psychologically and physiologically more difficult to perform mental tasks. However, there was no significant difference between vibration conditions for the overall TLX scores (Hancock et al., 2008[18]). The findings of the current study are consistent with the literature indicating that our participants experienced a higher level of MWL while they were exposed to WBV. 

The findings of the present study demonstrated that exposure to WBV could significantly reduce human performance compared to the unexposed condition. It is important to note that the effects of WBV on human performance requiring vision and hand-arm control are dependent on many parameters including vibration characteristics (i.e. frequency, amplitude and direction) seat characteristics (e.g. comfortability, existence of arm rest, dimensions, etc.), display and PC input device characteristics (e.g. size, brightness, sensitivity, etc.), task presentation characteristics (e.g. size, font, color, etc.) and environmental conditions. These parameters create differences amongst experiments, since the literature indicates inconsistent results. Studying cognitive responses to the three WBV levels (0.5, 0.81 and 1.12 m/s^2^, 3-7 Hz), Zamanian et al. (2014[49]) found that the reaction time of participants significantly increased in the divided attention test but remained unchanged in the sustained attention test compared to the control condition (Zamanian et al., 2014[49]). The study by Paddan et al. (2012[36]) showed that exposure to WBV might significantly increase the reaction time of participants but has no effect on the incorrect and missed responses compared to the unexposed condition. Additionally, it was demonstrated that the participants have different reaction times under different frequencies of vibration (Paddan et al., 2012[36]). Keijser et al. (2017[30]) found that exposure to vibration may potentially increase motor performance but has no effect on spatial detection measurement (Keijser et al., 2017[30]). Although most studies reported inconsistent findings, they concluded that an association exists between exposure to WBV and human performance. Inter-study and intra-study disagreement could be explained by the different methods used in these studies. Almost all studies applied different characteristics of WBV, different mental or cognitive tasks, as well as different performance measures. Most studies found a statistically significant relationship between exposure to WBV and decreasing human performance.

Generally, enhancing human performance and reducing mental loads have a direct relationship with road accidents (Marquart et al., 2015[34]; Papantoniou et al., 2017[37]). So that, the findings of the effect of WBV on human cognitive responses and perceived MWL have important implications for designing vehicles, where riders and drivers are exposed to WBV. Both factors directly could be improved by a well-design program for vehicles. Therefore, applying the results of this study might acquire three important achievements including reducing financial costs, reducing human costs and improving human well-being by reducing number of traffic accidents (Dimitriou and Poufinas, 2016[9]).

Evidence of reaction time and missed response impairments were also observed because of WBV exposure. These impairments may pose occupational safety issues, particularly when one considers the complexity of tasks that are generally attributed to machinery operators. Further study might be needed to find the causal relation between WBV exposure and occurrences of accidents or near misses.

Generally, the literature showed that exposure to WBV could affect cognitive responses and motor movements (Costa et al., 2014[5]; Hancock et al., 2008[18]). However, little evidence characterizes how this physical agent could impose these effects. Additionally, the detailed physiological/psychological mechanisms underlying the effects of WBV exposure on mental workload are still well not understood. Tracking tasks and choice reaction time task are psycho-motor and cognitive, respectively, and so, exposure to WBV could significantly affect them (Paddan et al., 2012[36]; Costa et al., 2014[5]). Corbridge and Griffin (1991[4]) observed that writing speed decreased and subjective ratings of writing difficulty increased with increasing vibration magnitude. Additionally, writing difficulty also increased with increasing duration of vibration (Corbridge and Griffin, 1991[4]). Costa et al. (2014[5]) observed that WBV exposure might significantly reduce motor skill performance and cognitive performance (Costa et al., 2014[5]). Generally, the vibration transmits through the body and potentially might affect all muscles (Mansfield and Griffin, 2000[33]). Then, the muscles try to keep the body stable by a wave of expansions and contractions (Huang and Griffin, 2006[23]; Ritzmann et al., 2010[39]). 

Totally, the prefrontal cortex region of brain plays a cardinal role in the cognitive activities (Fuster, 2001[15]). In another side, some limited evidence indicated that WBV exposure could increase the activation of the prefrontal region, resulting in extra oxygen and energy demands (Li et al., 2012[32]). So, it seems that the WBV by affecting this region of brain might interfere cognitive activities. Furthermore, the perception of higher levels of MWL while exposed to WBV could be the result of increased oxygen and energy demand from the prefrontal region where cognitive processes occur. Anyway, further investigations are needed to meet these unclear relationships.

## Conclusion

The present study observed that exposure to whole body vibration might adversely affect human performance and perceived mental workload. Additionally, the findings suggested that inclining the backrest angle up to 120 degrees has no effect on human responses to cognitive tasks. 

## Acknowledgement

This work was supported by Shiraz University of Medical Sciences (grant no. 11607).

## Figures and Tables

**Table 1:**
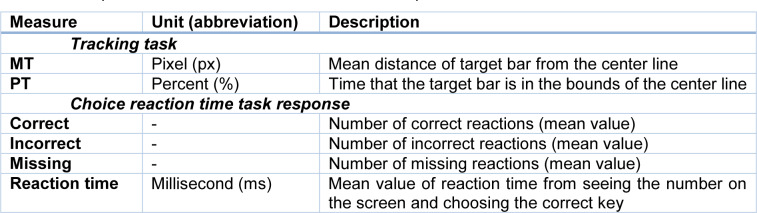
The performance measures obtained from computerized dual task

**Table 2:**
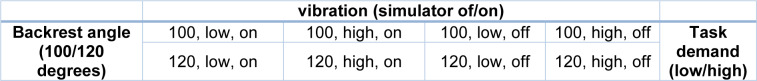
The matrix of three independent variables to make eight active tests

**Table 3:**
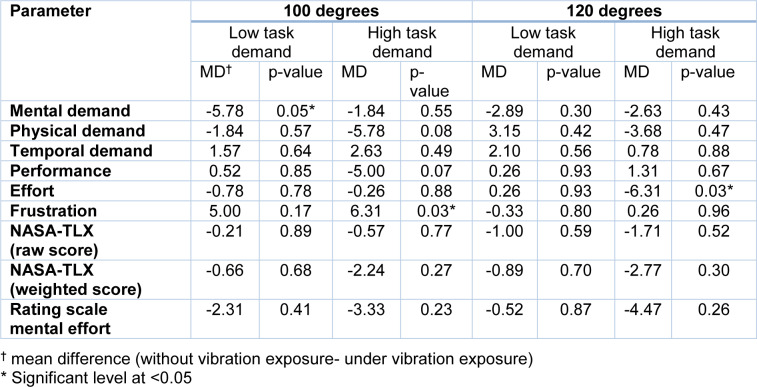
Comparison of perceived mental workload without/under vibration exposure

**Table 4:**
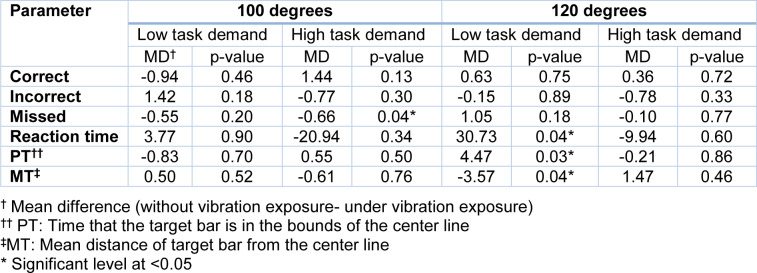
Comparison of performance under/without vibration exposure
